# Informative three-dimensional survey of cell/tissue architectures in thick paraffin sections by simple low-vacuum scanning electron microscopy

**DOI:** 10.1038/s41598-018-25840-8

**Published:** 2018-05-10

**Authors:** Akira Sawaguchi, Takeshi Kamimura, Atsushi Yamashita, Nobuyasu Takahashi, Kaori Ichikawa, Fumiyo Aoyama, Yujiro Asada

**Affiliations:** 1 Division of Ultrastructural Cell Biology, Department of Anatomy, Faculty of Medicine, University of Miyazaki, Miyazaki, 889-1692 Japan; 2 Hitachi High-Technologies Corporation, Tokyo, 105-8717 Japan; 3 Division of Pathophysiology, Department of Pathology, Faculty of Medicine, University of Miyazaki, Miyazaki, 889-1692 Japan

## Abstract

Recent advances in bio-medical research, such as the production of regenerative organs from stem cells, require three-dimensional analysis of cell/tissue architectures. High-resolution imaging by electron microscopy is the best way to elucidate complex cell/tissue architectures, but the conventional method requires a skillful and time-consuming preparation. The present study developed a three-dimensional survey method for assessing cell/tissue architectures in 30-µm-thick paraffin sections by taking advantage of backscattered electron imaging in a low-vacuum scanning electron microscope. As a result, in the kidney, the podocytes and their processes were clearly observed to cover the glomerulus. The 30 µm thickness facilitated an investigation on face-side (instead of sectioned) images of the epithelium and endothelium, which are rarely seen within conventional thin sections. In the testis, differentiated spermatozoa were three-dimensionally assembled in the middle of the seminiferous tubule. Further application to vascular-injury thrombus formation revealed the distinctive networks of fibrin fibres and platelets, capturing the erythrocytes into the thrombus. The four-segmented BSE detector provided topographic bird’s-eye images that allowed a three-dimensional understanding of the cell/tissue architectures at the electron-microscopic level. Here, we describe the precise procedures of this imaging method and provide representative electron micrographs of normal rat organs, experimental thrombus formation, and three-dimensionally cultured tumour cells.

## Introduction

One of the major goals of biological microscopy is to elucidate the structural evidence with which we can correlate functional activity. Recent advances in bio-medical researches, such as the production of regenerative organ from induced pluripotent stem (iPS) cells^1,2^ and the morphological changes induced by CRISPR/Cas9-mediated genome editing^3^, require three-dimensional analysis of those cell/tissue architectures. Scanning electron microscopy (SEM) provides three-dimensional information of specimen surfaces by collecting electrons reflected from the surface (backscattered electrons, BSE) and electrons forced out of the surface (secondary electrons, SE). Low-vacuum SEM allows for the BSE and/or SE imaging of non-conductive biological samples^4–7^ because the negative charge accumulations on the non-conductive materials can be eliminated with the positive ions in residual gas molecules^8,9^.

For light-microscopic examinations, paraffin wax continues to be the universal embedding medium for histological analysis, immunohistochemistry, and diagnostic histopathology, mainly because it is inexpensive and easily handled for sectioning. Electron microscopy of non-conductive paraffin sections takes advantages of BSE imaging in low-vacuum SEM^10,11^. However, the images obtained from such thin sections (5–10 µm in thickness) are basically two-dimensional, and it remains a challenge to reconstruct the missing third dimension by examining many sections of the three-dimensional cell/tissue architectures.

To address these problems, we applied 30-µm paraffin sections to BSE imaging in low-vacuum SEM (abbreviated to Thick PS-LvSEM). The application provided a simple three-dimensional survey method of the cell/tissue architectures embedded in the thick paraffin section, by which the missing third dimension could be viewed. Here we describe the precise procedures of this new method of three-dimensional imaging of thick paraffin sections for high-resolution cell/tissue architectures, accompanied by representative electron micrographs.

## Materials and Methods

### Sample preparation in anatomical experiments

**Figure 1:**
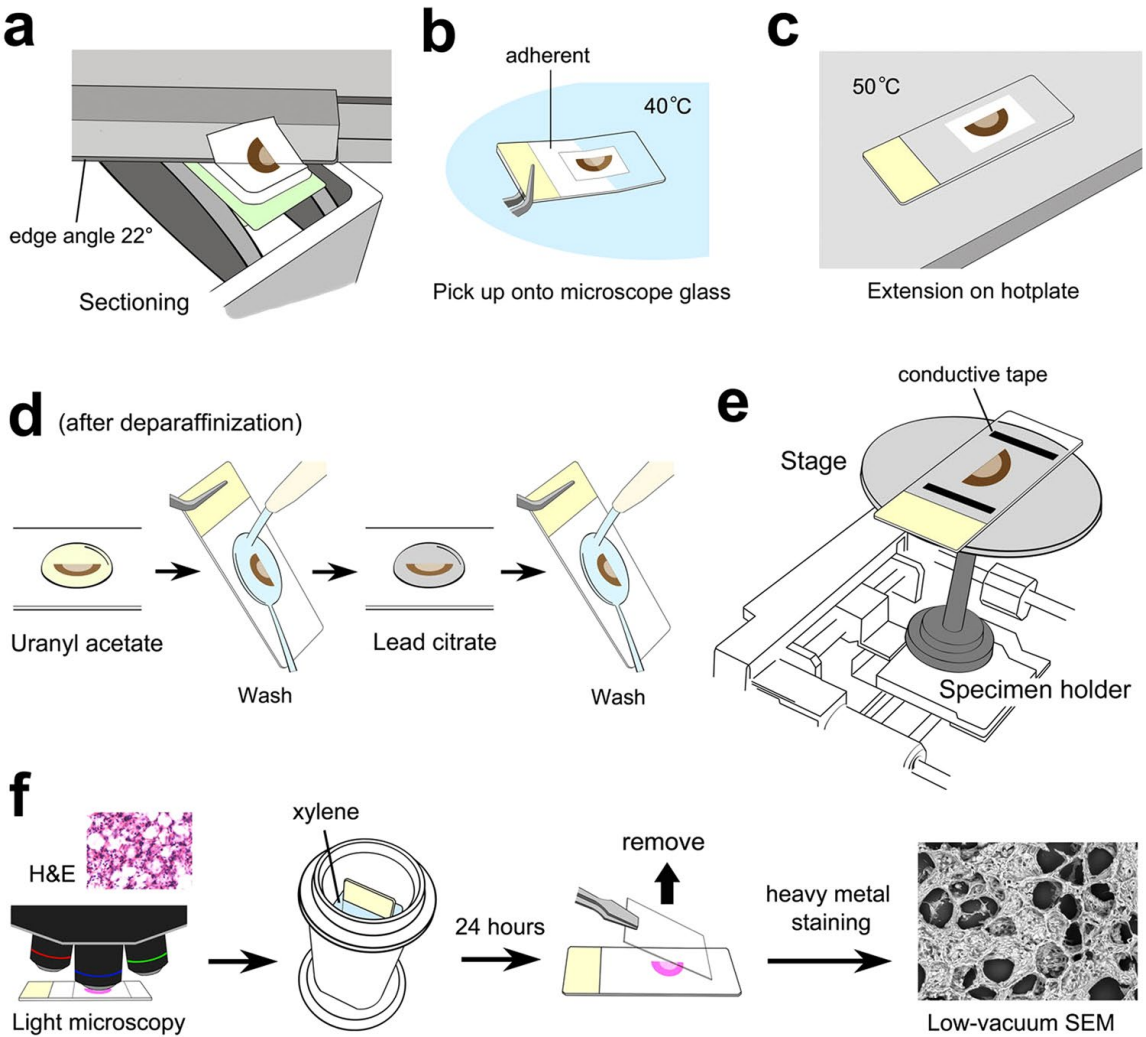
Sample preparation and anatomical findings by Thick PS-LvSEM. (**a**–**e**) Illustration of the basic workflow. (**a**) Sectioning. (**b**) Mounting onto adherent coating microscope slides. (**c**) Extension on a hot plate. (**d**) Staining with uranyl acetate followed by Reynold’s lead citrate. (**e**) Setting onto the wide stage of a specimen holder. (**f**) Illustrated procedure for the correlative light and electron microscopy.

Male Wistar rats (Kyudo, Kumamoto, Japan), 10 weeks old, were deeply anesthetized and then perfused with 4% paraformaldehyde in 0.1 M phosphate buffer (PB: pH 7.4) from the left ventricle of heart. The lung, kidney, trachea, esophagus, eye, pancreas, testis, spinal cord, auricles, and larynx were excised and further fixed by immersion in the above fixative for 2 hours at room temperature (RT). After washing in running tap water for 2 hours, the organs were dehydrated in a graded series of 50%, 70%, 80%, 90%, and 100% ethanol and cleared by xylene for 2 hours using an automatic tissue processor (TP 1020, Leica Microsystems, Wetzlar, Germany) to be embedded in paraffin (melting point 54–56 °C: Wako Pure Chemical Industries, Osaka, Japan) using a heated paraffin embedding station (HistoCore Arcadia H, Leica Microsystems GmbH). Then, 30-µm-thick sections were cut using a sliding microtome (Yamato Kohki Industrial, Saitama, Japan) with disposable stainless-steel knives (S-22, edge angle 22°: Feather safety razor, Osaka, Japan) (Fig. 1a). The use of a sharp 22° edge angle is critical to obtain thick sections over 20 µm in thickness. The sections were floated in a water bath (PS-125WH, Sakura Finetek Japan, Tokyo, Japan) at 40 °C to be mounted onto New Silane II-coated microscope slides (size 76 × 26 mm, thickness 1.0 mm: Muto Pure Chemical, Tokyo, Japan) (Fig. 1b), and they were extended on a slide warmer (PS-53, Sakura Finetek Japan) heated at 50 °C for 10 seconds (Fig. 1c). After drying within an incubator at 37 °C overnight, the sections were deparaffinized in xylene and rehydrated in a series of 100%, 90%, 70%, and 50% ethanol and running tap water (5 min each). The sections were stained with 1.0% uranyl acetate in 70% methanol (UA) for 5 min (Fig. 1d), washed with distilled water, and then stained with Reynolds’ lead citrate solution (LC) for 3 min. After washing with distilled water, the sections were dried for observation.

### Low-vacuum scanning electron microscopy

The sections were set onto the wide stage of specimen holder (Fig. 1e) using adhesive conductive tape, to be placed in a low-vacuum scanning electron microscope (LvSEM) (TM4000Plus or TM3030Plus, Hitachi High-Technologies, Tokyo, Japan). After evacuation of the specimen chamber a few minutes, the sections were observed under the electron beam accelerating voltage of 5 kV, 10 kV, or 15 kV. The observation angle was changed using the optional 30° or 45° tilt-holder. The LvSEM images were taken by the four-segmented BSE detector, and those four images were averaged into one image. Topographic images were reconstructed using Hitachi map 3D software (Hitachi High-Technologies), subtracting the difference among four-segmented shadow images in which the three-dimensional undulation had been reflected according to the segmented angle of each detector^12^.

### Correlating light and electron microscopy to verify ideal paraffin section thickness

A series of paraffin sections at 5 µm, 15 µm, 30 µm, and 50 µm thickness were cut and mounted onto microscope slides as described above. Deparaffinized and rehydrated sections were stained with Mayer’s hematoxylin for 3 min and exposed to running tap water at least for 3 hours to develop the colour. Next, the sections were stained with eosin diluted in 60% ethanol for 3 min, then rinsed in a graded series of 80%, 90%, and 100% ethanol for dehydration. After clearance in xylene, the sections were mounted within Malinol (Muto Pure Chemicals, Tokyo, Japan) covered with a NEO Micro cover glass (size 24 × 50 mm, thickness No.1 = 0.13–0.17 mm: Matsunami Glass, Osaka, Japan). After observations under a light microscope (BX51, Olympus, Tokyo, Japan) equipped with a digital camera (DP72, Olympus), the microscope slides were incubated in xylene for 18–24 hours at room temperature to remove the coverslips (Fig. 1f). The sections were rehydrated with a series of 100%, 90%, and 70% ethanol and distilled water (5 min each), then stained with UA and LC as described above.

### Comparison of paraffin section stainability for heavy metals

Deparaffinized and rehydrated 30-µm-thick paraffin sections of rat auricle and larynx were stained with UA alone for 5 min or LC alone for 3 min to be compared with the combined staining with UA and LC described above. As a control, platinum coating was performed using an ion sputter (E-1045, Hitachi High-Technologies) operating at 15 mA for 60 seconds (coating thickness = approximately 6.8 nm).

### Rabbit arterial thrombosis model by disturbed blood flow

Male Japanese white rabbits (Kyudo, Kumamoto, Japan) weighing approximately 2.5 kg were fed a conventional diet. Surgery proceeded under aseptic conditions and general anesthesia with the administration of intravenous pentobarbital (25 mg/kg). An angioplasty balloon catheter (diameter, 2.5 mm; length, 9 mm; QUANTUM, Boston Scientific, Galway, Ireland) was inserted via the carotid artery into the right femoral artery under fluoroscopic guidance. The catheter was inflated to 1.5 atm and retracted three times to induce smooth muscle cell (SMC)-rich atherosclerotic plaque in the right femoral artery^13,14^. Three weeks later, a 21-G needle (1 cm in length) was longitudinally placed along the ventral surface of the pre-treated femoral artery, and a 1–0 silk suture was tied at one point around both the artery and the needle, which was then removed to resume the blood flow^15^. The blood flow was reduced to approximately 25% of the initial level measured using a T106 transit flow meter (Transonic Systems Inc., NY, USA). These techniques were carried out under the utmost care to prevent traumatic endothelial injury. One hour later, the rabbits were injected with heparin (500 U/kg, i.v.) and euthanized with an overdose of pentobarbital (60 mg/kg, i.v.) to evaluate erosive damage to intima/neo-intima and thrombus formation. The animals were then perfused with 0.01 M phosphate-buffered saline (PBS) and 50 mL of 4% paraformaldehyde in 0.01 M PBS for the histological analyses. The excised femoral arteries were further fixed in 4% paraformaldehyde for 12 hours at 4 °C and dehydrated specimens were longitudinally embedded in paraffin to be cut into 5 or 30 µm in thickness as described above. Target imaging of thrombus formation was performed by pre-evaluation under light microscopy (with H&E-stained 5 µm-thin sections) and following thick sectioning from the same paraffin block for Thick PS-LvSEM.

### Xenograft of SUIT-58 pancreas cancer cell line

A cancer cell line, SUIT-58^16^, was established from a metastatic liver tumour that originated from pancreas cancer. The SUIT-58 cells were cultured in a mixture of RPMI1640 (Nissui, Tokyo, Japan) and Ham’s F12 (Nissui, Tokyo, Japan) supplemented with 10% heat-inactivated fetal bovine serum in a humidified atmosphere of 5% CO_2_ at 37 °C. After cultivation, SUIT-58 cells were trypsinized with 0.25% trypsin and 0.02% ethylenediamine tetraacetic acid (Sigma-Aldrich, St. Louis, Missouri, USA), and centrifuged immediately at 1,000 g for 5 min. Then, resuspended cells (1 × 10^5^ cells/0.2 ml PBS) were made into a xenograft prepared by subcutaneous injection at the abdominal flank of male 6-weeks-old BALB/c athymic mice (Kyudo, Kumamoto, Japan). Eight weeks later, the xenograft was harvested under general anesthesia, and fixed by immersion into 4% paraformaldehyde in 0.1 M PB for 30 min. The fixed xenografts were processed into 30-µm-thick paraffin sections as described in anatomical experiments.

### Three-dimensional culture of SUIT-58 cell line

Three-dimensional culture of the SUIT-58 cell was performed within type I collagen gel (Cellmatrix I-A: Nitta Gelatin, Osaka, Japan) under air-exposure to promote cell growth^17^. First, prior to the cultivation, 1 ml of collagen gel was poured into each well of a Cell Culture Insert (6-well format, pore size 3.0 µm: Falcon, New York, USA) combined with a microplate (Iwaki, Shizuoka, Japan), and solidified within an incubator at 37 °C for 30 min. Then, the SUIT-58 cell suspension (1 × 10^3^ cells/2 ml) was poured over the solid acellular layer and covered with 1 ml of collagen gel. After solidification of the additional collagen gel layer, 4.0 ml of the culture media was poured into the outer culture plate. The culture medium was changed in every other day, and approximately eight weeks later, the cultivated SUIT-58 cells among the collagen gel layers were fixed with 4% paraformaldehyde in 0.1 M PB for 1 hour and processed for paraffin embedment.

All animal procedures were carried out under protocols approved by the University of Miyazaki Animal Research Committee, in accordance with international guiding principles for biomedical research involving animals.

### Data availability statement

The raw light and electron micrographs are available at 10.6084/m9.figshare.5640088.

## Results and Discussion

### Three-dimensional cell/tissue architectures in normal rat organs

**Figure 2:**
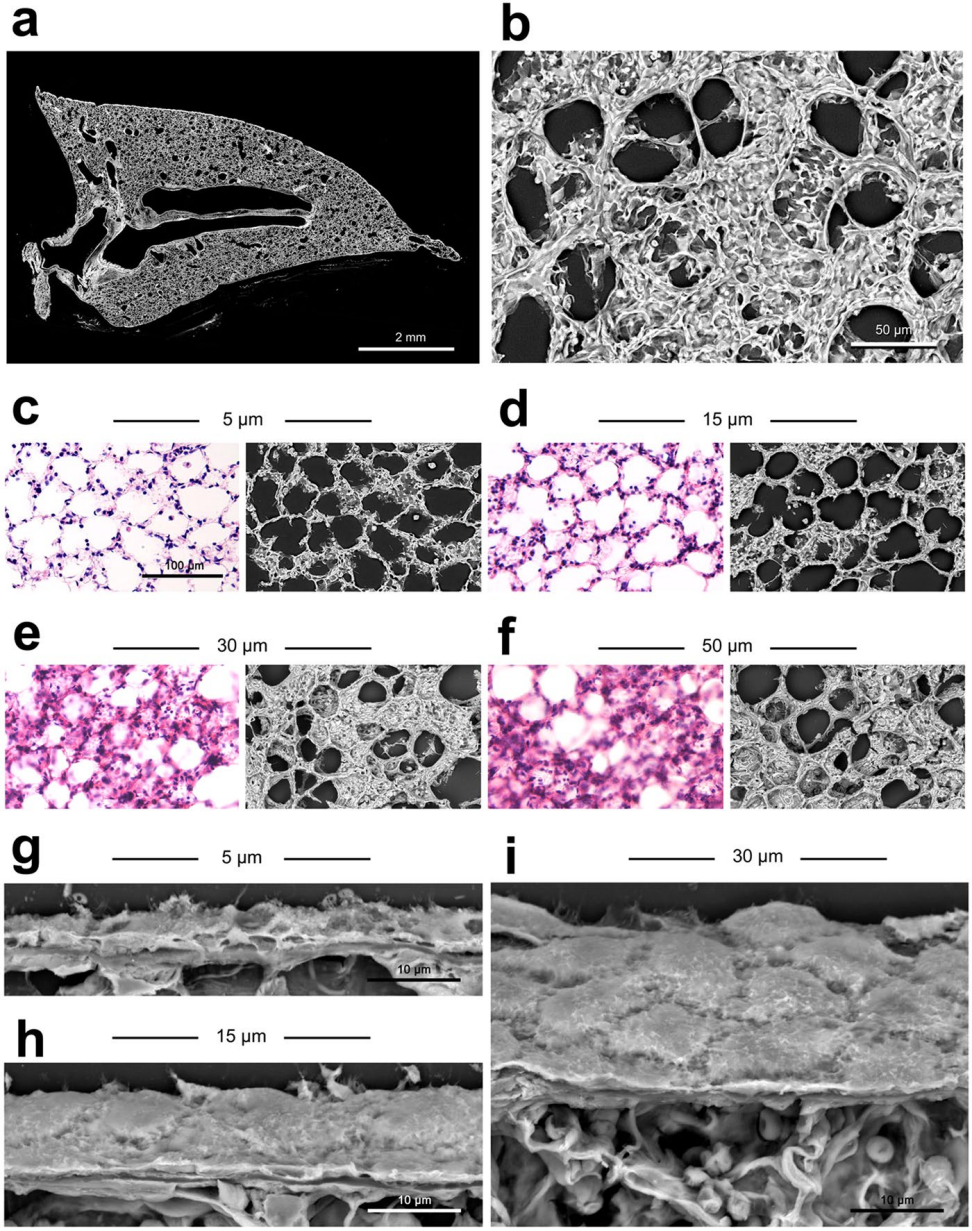
Preliminary examinations to determine the deal thickness of paraffin sections. (**a**,**b**) Lung. Montage image of whole section (**a**) and high-power view of the pulmonary alveoli with its wall face image (**b**). (**c**–**f**) Correlative light (H&E staining) and Thick PS-LvSEM microscopy of rat lung paraffin sections in a series of 5, 15, 30, and 50 µm thickness. The sectioned features of alveolar septum are dominant in the 5 µm (**c**) and 15 µm (**d**) sections. On the other hand, the wall-face features occupy more than half the area in the 30 µm (**e**) and 50 µm (**f**) sections. Note the unavoidable cracks in the 50 µm sections. (**g**–**i**) Comparison of oblique-section images of pulmonary pleura in 5 µm (**g**), 15 µm (**h**), and 30 µm (**i**) sections.

First, the present approach enabled us to survey whole section on the centimetre scale, as shown in a representative montage of rat lung (Fig. 2a), in contrast to the millimeter-scale sample size of conventional electron microscopy. Second, following the wide-range survey, higher magnification demonstrated fine structures of the pulmonary alveoli (Fig. 2b). It should be emphasized that 30-µm-thick section disclosed the wall-face of pulmonary alveoli as well as the common sectional images of alveolar septum. In our preliminary experiments (Fig. 2c–i), 30-µm-thick sections were the most informative among the 5-, 15-, 30-, and 50-µm sections because of the unavoidable cracks found in the 50-µm-thick sections (Fig. 2f). As illustrated in Fig. 1f, the simple procedure of correlative light and electron microscopy was useful for the comparative demonstration between histochemical staining and electron-microscopic findings (Fig. 2c–f). Biological cell diameters occasionally exceed 30 µm, but oblique section images at 30 µm thickness (Fig. 2i) further exceeded this size and were rather informative to reveal three-dimensional cell/tissue architectures in Thick PS-LvSEM.

**Figure 3:**
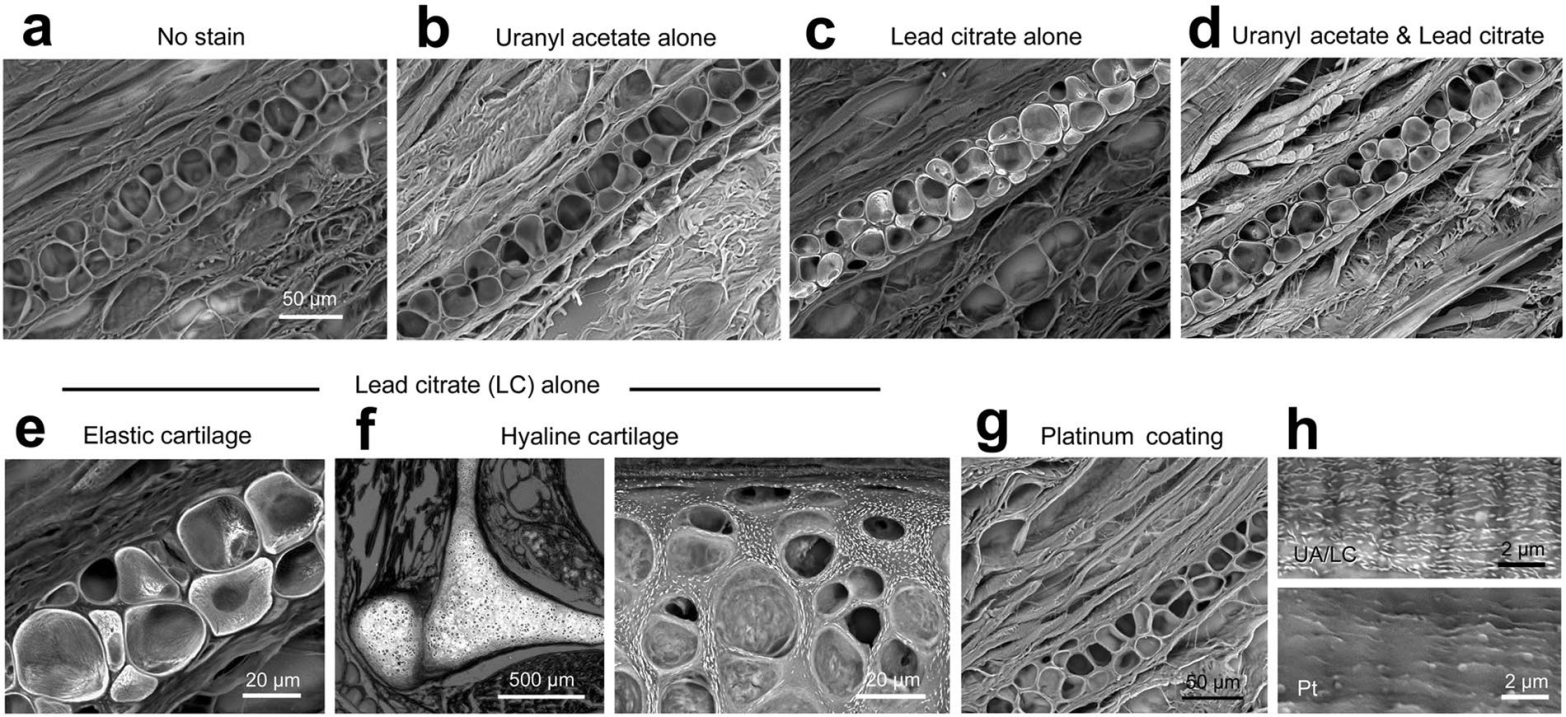
Preliminary examinations to determine the stainability of 30 µm paraffin sections. (**a**–**e**,**g**,**h**) Rat auricle and (**f**) larynx. (**a**) No stain. (**b**) 1% uranyl acetate in 70% methanol (UA) alone. (**c**) Reynolds’ lead citrate solution (LC) alone. (**d**) UA and LC. (**e**,**f**) High-power view of elastic cartilage (**e**), and hyaline cartilage (**f**) in larynx stained with LC alone. Note the remarkable LC-stainability of the cartilage, as shown in the high-power view of hyaline matrix (right in **f**). (**g**) Platinum coating. (**h**) Comparison between UA/LC staining (upper) and platinum coating (lower) on skeletal muscle fibre. The skeletal muscle striation is significantly masked by the platinum coating.

Another preliminary experiment reappraised the conventional heavy metal staining with uranyl acetate and lead citrate, which is routinely used for ultrathin sections of epoxy resin in transmission electron microscopy (Fig. 3a–d). Interestingly, the lead citrate staining exhibited a strong affinity to the elastic cartilage in the auricle (Fig. 3e) and the hyaline cartilage in the larynx (Fig. 3f), implying the histochemical localization of the cartilage constituent. Alternative to heavy metal staining, heavy metal coating is also routinely performed in the conventional SEM to increase both image contrast and electron conductivity (Fig. 3g). However, in our verification, the skeletal muscle striation was significantly masked by the platinum coating (approximately 6.8 nm in thickness: Fig. 3h), inferior to the result of heavy metal staining.

**Figure 4:**
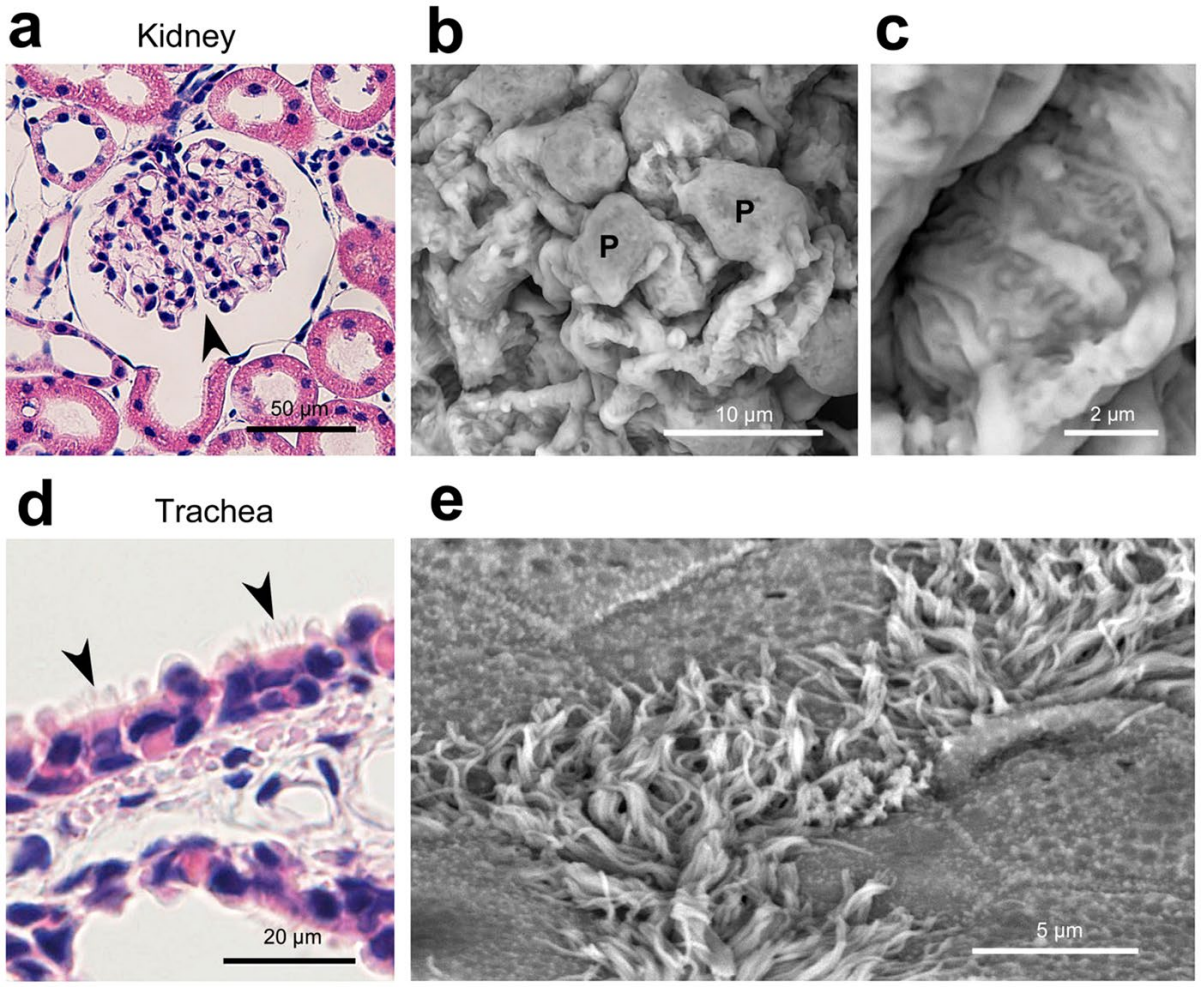
Representative micrographs of rat kidney and trachea. Electron micrographs were taken from 30 µm sections. (**a**–**c**) Kidney. (**a**) Overview of the glomerulus (arrowhead) within the renal corpuscle. (**b**) Podocytes (P) covering the glomerular capillaries. (**c**) High-power view demonstrates the engaged processes. (**d**,**e**) Trachea. Note the ciliated epithelial cells (arrowheads) distinguished by Thick PS-LvSEM.

**Figure 5:**
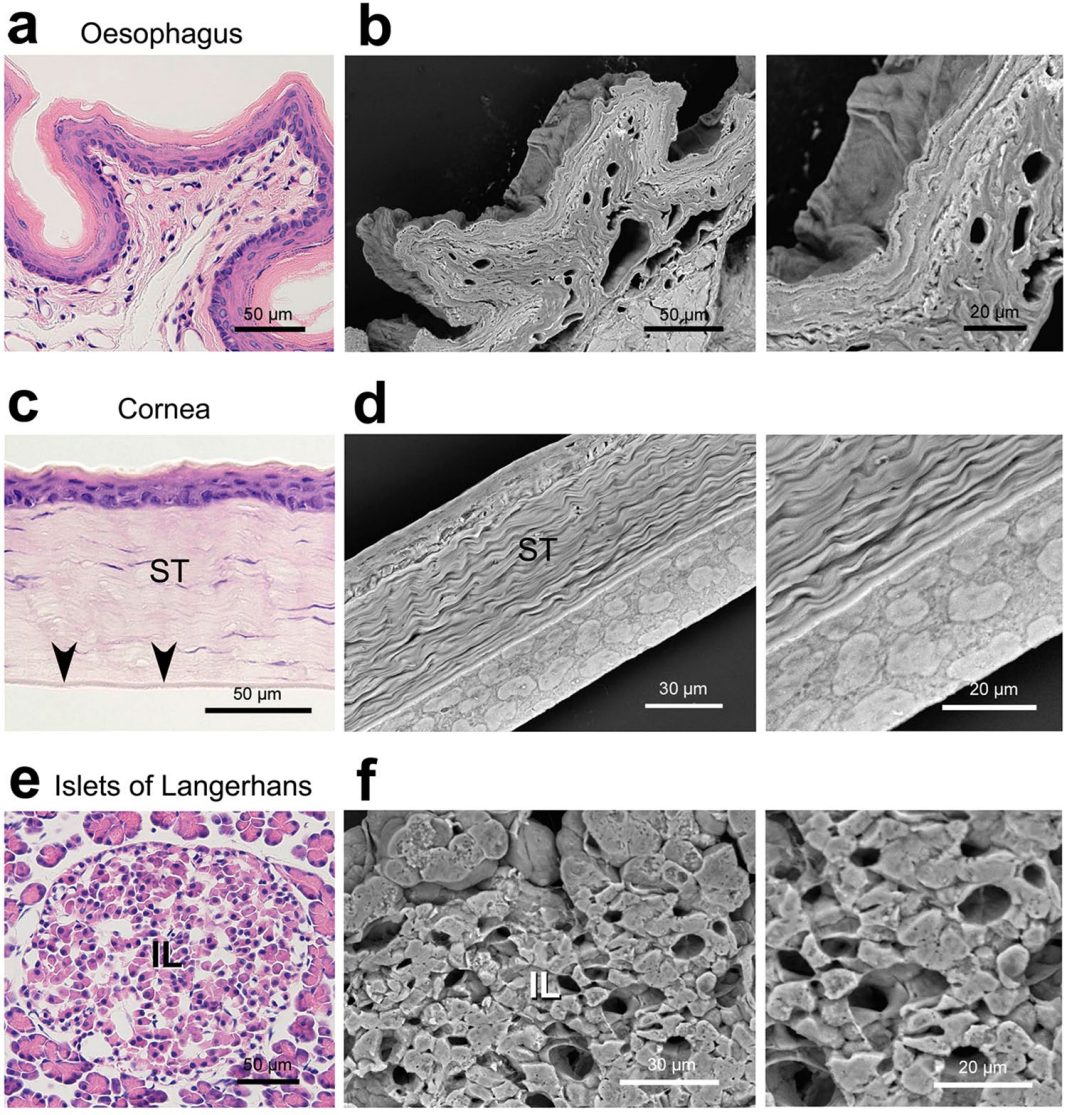
Representative micrographs of rat oesophagus, cornea, and pancreas. Electron micrographs were taken from 30 µm sections. (**a**,**b**) Oesophagus. Thick PS-LvSEM demonstrates the multiple layers of stratified epithelium. (**c**,**d**) Cornea. The face image of endothelium, rarely seen in 5 µm sections (arrowheads in **c**), is clearly shown in the 30 µm section. Note the collagen fibres in its stroma (ST). (**e**,**f**) Islets of Langerhans (IL) in the pancreas. The capillary network is seen, a characteristic of endocrine organs.

**Figure 6:**
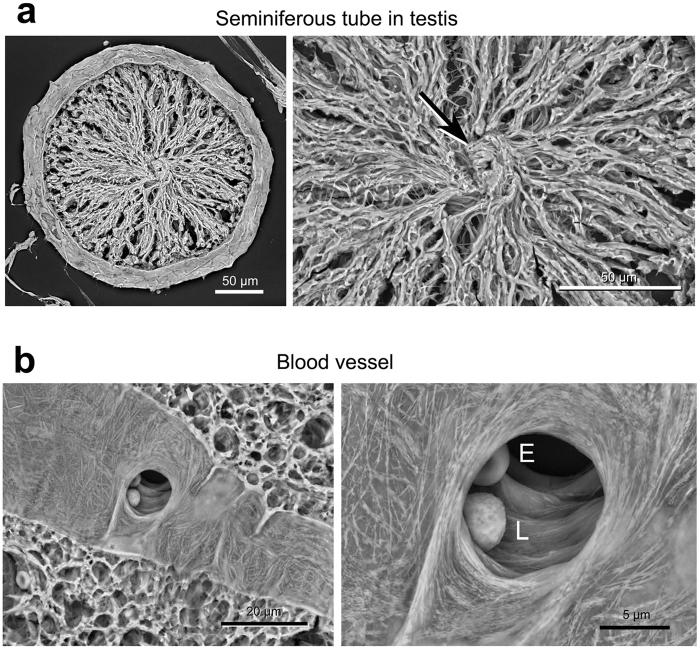
Representative micrograph of rat testis and blood vessel. Electron micrographs were taken from 30 µm sections. (**a**) Seminiferous tubule in the testis. Differentiated sperm cells are assembled in the centre (arrow). (**b**) A blood vessel in the spinal cord. Note the vascular endothelium and its bifurcation with erythrocyte (E) and leukocyte (L).

By means of the established protocol, we first investigated representative cell/tissue architectures in normal rat organs embedded in 30-µm-thick paraffin sections (Figs 4–6). In the kidney, the podocytes and their processes were clearly observed to cover the glomerulus (Fig. 4a–c). Next, in the trachea, the Thick PS-LvSEM distinguished a number of the cilia protruding from the respiratory epithelium (Fig. 4d,e). It is noteworthy that the thickness facilitated investigation on a face-side (instead of sectioned) images of the epithelium (Fig. 5a,b) and endothelium (Fig. 5c,d), which is rarely seen within the thin section. Higher magnification revealed fine structures of the stratified structure in the oesophagus (Fig. 5b) and the collagen fibrils in the stroma of the cornea (Fig. 5d). The islets of Langerhans exhibited complicated capillary structures among the endocrine cells, a characteristic of endocrine organs (Fig. 5e,f). Interestingly, in the testis, differentiated sperm cells were three-dimensionally assembled in the middle of seminiferous tubule (Fig. 6a). Moreover, the Thick PS-LvSEM provided a wall-face image of the blood vessel wall, including its three-dimensional bifurcation on occasion (Fig. 6b).

### Application to pathological experiments

**Figure 7:**
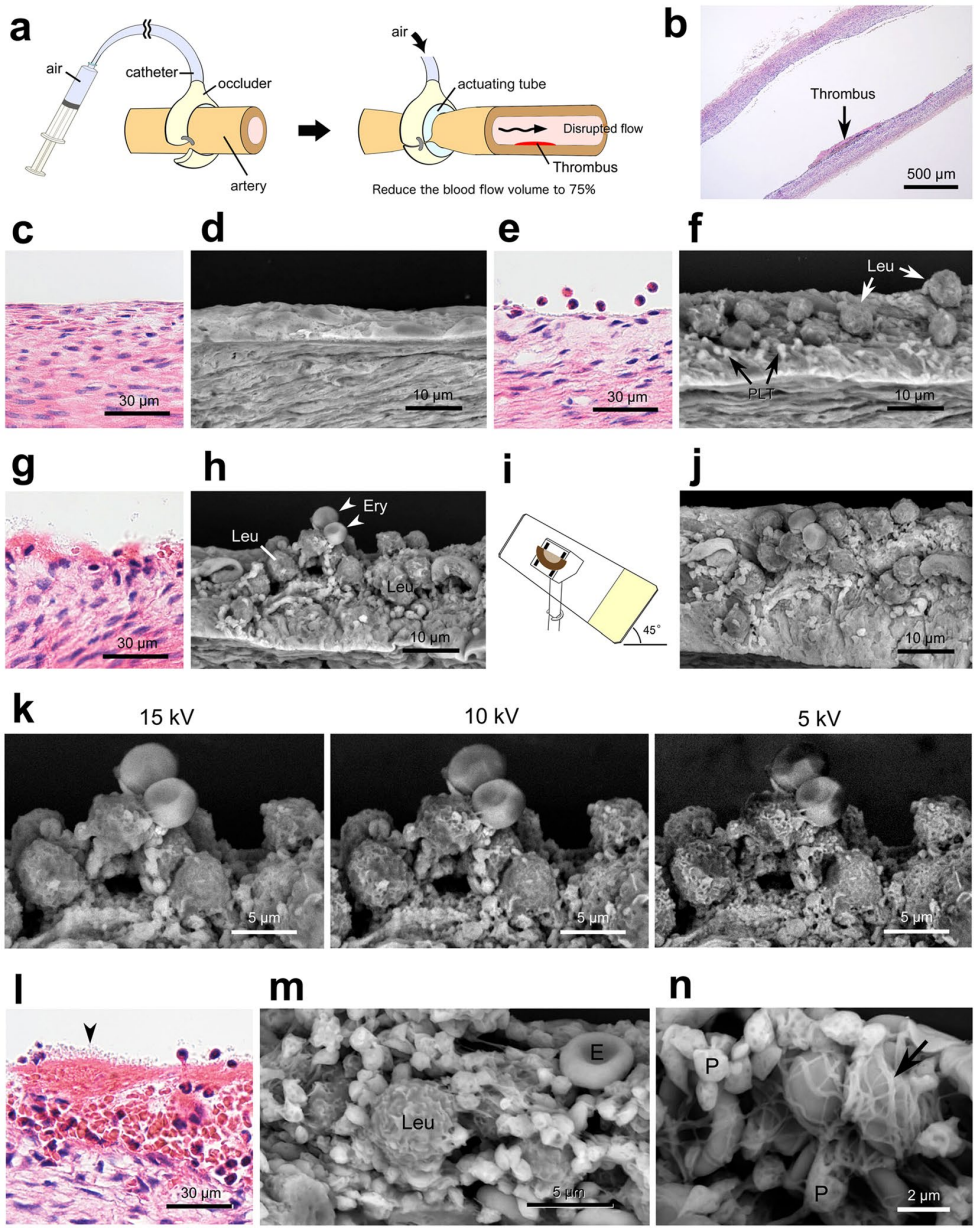
Application of Thick PS-LvSEM to pathological experiments. (**a**–**n**) An experimental model of thrombus formation induced by disturbing the blood flow in the rabbit femoral artery. All images were taken from 30 µm sections, except the light micrographs from 5 µm sections with H&E staining (**b**,**c**,**e**,**g**,**l**). (**a**) Illustration of the arterial blood flow disturbance. (**b**) Overview of the mural thrombus formation. (**c**,**d**) Normal endothelium. (**e**,**f**) Adhesion of leukocytes (Leu) and platelets (PLT) on the erosive endothelium. (**g**–**k**) Aggregations of leukocytes (Leu), erythrocytes (Ery), and platelets, attaching to the sub-endothelium exposed in close proximity to the thrombus. (**i**) Illustrated use of the optional 45° tilt-holder. (**j**) Tilted top view of the aggregation. (**k**) Comparison of accelerating voltages. Note the higher signal-to-noise ratio at 15 kV, and the surface configuration revealed at 5 kV. (**l**–**n**) Core portion of the mural thrombus consisting of erythrocytes (E), leukocytes (Leu), platelets (P, and arrowhead in **l**), and fibrin fibres (arrow). (**n**) Note the network of fibrin fibres and platelets capturing the erythrocytes.

We applied the Thick PS-LvSEM to pathological experiments to vascular injury thrombus formation (Fig. 7a–n). Acute myocardial infarction is triggered by the disruption of coronary atherosclerotic plaques and thrombus formation^18^. The present experimental thrombus was induced by blood flow disturbance in the rabbit femoral artery following the previous induction of smooth muscle cell-rich neointima^13,14^ (Fig. 7a,b). As a result, in contrast to normal endothelium (Fig. 7c,d), a slight endothelial lesion demonstrated scattered adhesions of the leukocytes accompanied by platelets in the post-stenotic region (Fig. 7e,f). Adjacent to the thrombus, further aggregations consisted of leukocytes, erythrocytes, and a number of platelets (Fig. 7g,h). The optional 45° tilt-holder provided a top view of the aggregation covering the sub-endothelial connective tissue exposed by severe detachments of the endothelial cells (Fig. 7i,j). We simultaneously examined the ideal accelerating voltage for the Thick PS-LvSEM and concluded that a higher accelerating voltage (15 kV) yielded superior quality, with a high signal-to-noise ratio (Fig. 7k). On the other hand, the lower accelerating voltage (5 kV) demonstrated the detail of the surface configuration because the origin of BSE information was confined to the surface layer on account of the weak electron beam^19^. It is therefore important to select the accelerating voltage that is ideal for the objective. Finally, the Thick PS-LvSEM revealed the distinctive networks of fibrin fibres and platelets, capturing the erythrocytes in the main mural thrombus (Fig. 7l–n). These findings were invisible by light microscopy and were undetectable within the thin (less than 10 µm) sections because the diameter of leukocytes (neutrophils = 12–15 µm) exceeds that thickness.

### Application to oncological experiments

**Figure 8:**
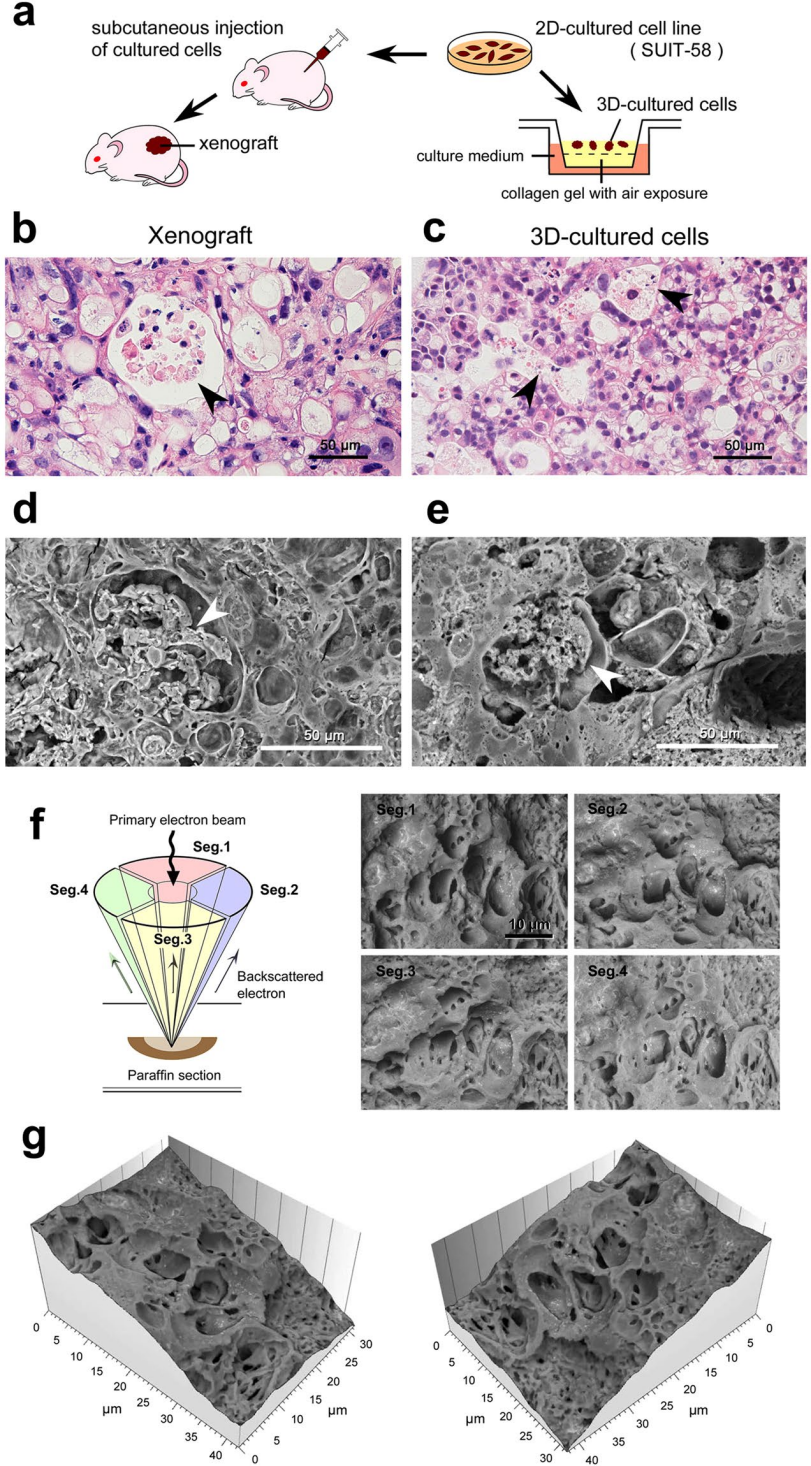
Application of Thick PS-LvSEM to oncological experiments using SUIT-58 cell line. (**a**) Illustration of the comparative experiments between xenograft and three-dimensionally cultured cells. (**b**,**c**) Light micrographs of H&E stained 5 µm sections and (**d**–**g**) Thick PS-LvSEM micrographs of 30 µm sections. Note the similar profiles of vacuole formation enclosing necrotic cell debris (arrowheads). (**f**) Illustration of the four-segmented BSE detectors (left) and collected micrographs by each detector (right). (**g**) Topographic images reconstructed from the raw micrographs of the four-segmented BSE images.

We next explored the Thick PS-LvSEM to oncological investigation with a special reference to new establishment of tumour cell line that requires morphological comparison between *in vivo* xenografts and *in vitro* (usually two-dimensionally) cultured cells (Fig. 8a–e). The examined pancreas tumor cell line, SUIT-58^15^, represents the characteristics of moderately differentiated adenocarcinoma. In this study, we applied a three-dimensional culture system^17^ to make the most use of the Thick PS-LvSEM and confirmed the similarity between its xenografts and the three-dimensionally cultured cells (Fig. 8b,c). Beyond the light-microscopic comparison, the Thick PS-LvSEM demonstrated the ultrastructure of vacuoles enclosing necrotic cell debris (Fig. 8d,e), and also elucidated the three-dimensional undulation by means of the four segmented BSE detector^12^ (Fig. 8f). Those four segmented BSE images were processed into a topographic bird’s-eye image^20^ (Fig. 8g and Supplementary Video S1), leading to a three-dimensional understanding of the cell/tissue architectures at the electron microscopic level.

Formalin-fixed, paraffin-embedded tissue blocks are the most common form of tissue stored in biorepositories of clinical tissue specimens^21,22^. Clinically annotated specimens are valuable to identify prognostic cancer-relating biomarkers in retrospective analyses^23,24^. Indeed, multiplexed fluorescence microscopy has been applied to quantitative, single-cell analysis of colorectal cancer tissue^25^. Most recently, to overcome the limited resolution, the super-resolution fluorescence microscopy has also been applied to formalin-fixed paraffin-embedded human rectal^26^ and breast cancer tissues^27^ for pathological investigations.

High-resolution imaging by electron microscopy remains superior to elucidate the complex cell/tissue architectures. However, it is generally thought that the electron microscopy is a troublesome technique requiring skillful and time-consuming sample preparation as well as complicated operation of the electron microscope. In this context, it could be noteworthy that the present sample preparation for Thick PS-LvSEM requires no special equipment or techniques because it follows conventional protocols, except for the thickness of the paraffin sections, for light microscopy and heavy metal staining for electron microscopy.

BSE imaging has been widely applied to three-dimensional imaging of serial block-face (SBF) SEM, focused ion beam (FIB) SEM, and automated tape-collecting ultramicrotome (ATUM) SEM^28^, but those techniques require time-consuming sample preparation and image acquisition (several hours)^29^. In addition, their maximum specimen widths are restricted to 100 µm (FIB-SEM), 1 mm (SBF-SEM), and 3 mm (ATUM-SEM)^29^, much smaller than the centimetre scale for the Thick PS-LvSEM. The Thick PS-LvSEM method provides instant overview and screening of cell/tissue architectures that are invisible by light microscopy.

In conclusion, our Thick PS-LvSEM will remove, or at least lower, those hurdles for life scientists and bridge the gap between light and conventional electron microscopy. It is also important that the paraffin-embedded samples are semi-permanent and that their use in Thick PS-LvSEM enables a retrospective investigation of valuable old samples (prepared decades ago)^30^. Further application of Thick PS-LvSEM is expected to solve recent bio-medical challenges, such as the production of regenerative organs using iPS cells^1,2^, by visualizing three-dimensional cell/tissue architectures embedded in thick paraffin sections.

## Electronic supplementary material

Supplementary Video S1
